# Prolonged persistence of canine distemper virus RNA, and virus isolation in naturally infected shelter dogs

**DOI:** 10.1371/journal.pone.0280186

**Published:** 2023-01-20

**Authors:** Carolyn Allen, Alexandre Ellis, Ruibin Liang, Ailam Lim, Sandra Newbury

**Affiliations:** 1 Department of Medical Sciences, Shelter Medicine Program, University of Wisconsin, Madison, Wisconsin, United States of America; 2 Wisconsin Veterinary Diagnostic Laboratory, Virology, University of Wisconsin, Madison, Wisconsin, United States of America; Taif University, SAUDI ARABIA

## Abstract

Canine distemper virus remains an important source of morbidity and mortality in animal shelters. RT-PCR is commonly used to aid diagnosis and has been used to monitor dogs testing positive over time to gauge the end of infectious potential. Many dogs excrete viral RNA for prolonged periods which has complicated disease management. The goal of this retrospective study was to describe the duration and characteristics of viral RNA excretion in shelter dogs with naturally occurring CDV and investigate the relationship between that viral RNA excretion and infectious potential using virus isolation data. Records from 98 different humane organizations with suspect CDV were reviewed. A total of 5,920 dogs were tested with 1,393; 4,452; and 75 found to be positive, negative, or suspect on RT-PCR respectively. The median duration of a positive test was 34 days (n = 325), and 25% (82/325) of the dogs still excreting viral RNA after 62 days of monitoring. Virus isolation was performed in six dogs who were RT-PCR positive for > 60 days. Infectious virus was isolated only within the first two weeks of monitoring at or around the peak viral RNA excretion (as detected by the lowest cycle threshold) reported for each dog. Our findings suggest that peak viral RNA excretion and the days surrounding it might be used as a functional marker to gauge the end of infectious risk. Clarifying the earliest point in time when dogs testing positive for canine distemper by RT-PCR can be considered non-contagious will improve welfare and lifesaving potential of shelters by enabling recovered dogs to be cleared more quickly for live release outcomes.

## Introduction

Canine Distemper Virus (CDV) is an enveloped RNA virus of the family *Paramyxoviridae* and genus *Morbillivirus* characterized by a large host-range of terrestrial and non-terrestrial carnivores including family *Canidae* [1, 2]. CDV has been in existence for centuries with documentation as early as the 1735 CE [1]. Within genus Morbillivirus, CDV is predated only by the other infamous members, Rinderpest Virus and Measles Virus (MV). Evidence from historical accounts, teeth found in archeological sites and gene sequencing suggest CDV is likely the result of adaptation of MV to dogs making MV the likely parent virus of CDV [1].

Clinical signs of CD (Canine Distemper) result from both viral induced immunosuppression and the direct action of the virus on epithelial cells of the respiratory tract, gastrointestinal tract, and skin. Viral effects may progress to the nervous system [3, 4]. CDV initially infects and kills lymphocytes and mononuclear cells causing significant immunosuppression by binding to the signaling lymphocyte activation molecule (SLAM; CD150) receptor [5]. CDV can cause serious disease by binding to the Nectin-4 receptor while infecting epithelial cells in multiple systems or neuronal cells [6, 7].

While many lifesaving advances have been made in prevention and response, several outbreaks of CD in animal shelters are still investigated by the authors each year. CD remains a substantial cause of morbidity and mortality in animal shelters and communities that experience barriers to accessing veterinary care [8]. Because the clinical presentation of CD is highly variable and mimics other more benign shelter disease, a range of diagnostic testing is utilized to confirm the presence of the pathogen. The most sensitive and widely available diagnostic tests for CDV are commercial real time reverse transcription-polymerase chain reaction (RT-PCR) and real-time quantitative reverse transcription-polymerase chain reaction (RT-qPCR) assays [9, 10]. RT-PCR helps greatly to identify possible cases but many dogs continue to test positive on RT-PCR over prolonged periods of time creating welfare risks from extremely long shelter stays and uncertainty about when clinically recovered dogs can be considered safe and non-infectious.

Studies of other RNA viruses, such as MV and Severe Acute Respiratory Syndrome Coronavirus-2 (SARS-CoV-2), have suggested that patterns of prolonged excretion of viral RNA demonstrated by RT-PCR may not be indicative of continued infectious potential and that a more accurate marker of the end of infectious risk might be found [11, 12].

The goals of this study were to describe the duration and characteristics of viral RNA excretion in dogs testing positive in animal shelters via RT-PCR for CDV and investigate infectious potential over time through virus isolation (VI) of samples from naturally infected dogs who were positive on RT-PCR for prolonged periods of time. This study demonstrates, through extended longitudinal RT-PCR testing, that prolonged shedding of viral RNA is common but also demonstrates through VI that positive RT-PCR results do not always correspond to infectious potential. Comparison of VI and RT-PCR in naturally infected dogs with CDV, suggests a new marker for identifying the end of the infectious phase that could save resources and lives, improve welfare, and reduce prolonged lengths of stay for shelter dogs with CD.

## Material and methods

### Case inclusion for RT-PCR

Records of 5,920 dogs from 98 different animal welfare organizations in the United States who were tested for CDV by the University of Wisconsin Shelter Medicine Program between May 2014 and June 2020 were reviewed. Dogs were tested at the discretion of a veterinarian either as part of a shelter outbreak response or due to suspicion of disease in an individual animal. Permits or approvals for this retrospective study of clinical data were not required. Samples were gathered and tested in a clinical context to drive diagnosis and decision making for individual animals and groups of animals during clinical consultation, intervention, and response in disease outbreak situations.

RT-PCR testing for CDV was performed at the Wisconsin Veterinary Diagnostic Laboratory (WVDL), Madison WI. Data regarding age (often estimated), vaccination history, and presence or history of clinical signs in individual dogs, were collected at the time of initial testing. Interval between testing was variable and based upon financial resources, capacity for sample collection, and outbreak or case management strategies (S1 Dataset).

### Specimen collection

Specimens were collected by veterinarians and/or trained shelter staff. Diagnostic specimens were collected by swabbing the inside of one or both nares and/or the deep pharyngeal region using sterile swabs that were wrapped in pairs. The sterile polyester swabs used were kept unexposed to the environment until the moment of sample collection and gloves were changed between individual animals. Immediately after collection, swabs were placed in 3 mL of universal viral transport media (Becton, Dickinson and Company, Franklin Lakes, NJ) or 1 mL of sterile phosphate buffered saline in a sterile container. Samples were shipped overnight on wet ice to the WVDL and stored at 4°C until testing was completed. Samples kept for viral isolation were then stored in -80°C until virus isolation was performed.

### RNA extraction and RT-PCR

RNA extraction and RT-PCR were performed using an assay as previously described [10]. Briefly, nasal swabs were vortexed in the transport media, and 50 μL of the media was transferred for nucleic acid extraction using the MagMAX-96 Viral RNA Isolation Kit (Applied Biosystems, Foster City, CA) as described by the manufacturer. An in-house internal control (unpublished) was spiked in the extraction to monitor for PCR inhibition. Eight μL of the extracted nucleic acid was used to perform WVDL standard diagnostic RT-PCR assay for CDV, set up as a multiplex with an in-house internal control assay. The CDV RT-PCR assay utilized the published sequences for primers and probe targeting 87 bp of the CDV N protein-coding region [10]. A modification to the RT-PCR assay was made where the hydrolysis probe was labeled with fluorescein amidites (FAM) and a black hole quencher and the Path ID Multiplex One-Step RT-PCR kit (Applied Biosystems, Foster City, CA) was used. The 25 μL of RT-PCR master mix consisted of 1x Multiplex RT-PCR buffer, 1x Multiplex enzyme mix provided in the kit, 160 nM each of CDV forward and reverse primer (Integrated DNA Technologies, Coralville, IA), 80 nM of CDV hydrolysis probe (Biosearch Technologies, Novato, CA), 16 nM each of forward and reverse primer (Integrated DNA Technologies, Coralville, IA) and 8 nM of hydrolysis probe (Biosearch Technologies, Novato, CA) for in-house internal control, and 8 μL of the extracted nucleic acids. RT-PCR was performed in the ABI 7500 Real-time PCR System (Applied Biosystems, Foster City, CA) for 1 cycle of 50°C for 10 minutes, 1 cycle of 95°C for 10 minutes, and 40 cycles of 95°C for 15 seconds and 60°C for 1 minute. Any sample with an amplification plot that rose above the threshold and produced a cycle threshold (Ct) value less than 40 was considered positive. A sample that had a low level amplification plot that never reached the threshold was reported as suspect. Sample with no rise in the amplification plot was considered negative.

### Case inclusion for virus isolation

Two criteria were used for selecting dogs for virus isolation testing. First, in order to evaluate infectious potential in dogs with a long duration of positive results dogs were sorted to those with a series of positive test results that occurred over a span of time that was sixty days duration or more from the time of the first test. Second, to evaluate infectious potential at the early stages of infection, cases were filtered to include only dogs with low initial or early Ct results (≤26.5) that suggested testing may have begun early in infection. Of the dogs that fit these two criteria, dogs were selected for VI testing if appropriate samples were still available for virus isolation. Six dogs met all three criteria. These six dogs all had tested positive at least twice and eventually tested negative twice.

### Virus Isolation

SLAM expressing Vero cells (Vero.DogSLAMtag, or VDS) were grown and maintained in Dulbecco modified Eagle medium (Gibco, Waltham, MA) supplemented with 5% heat inactivated fetal bovine serum (Hyclone, Logan UT), 1% v/v penicillin streptomycin solution (Invitrogen, Carlsbad, CA), and 0.4 mg/mL of G418 (Promega, Madison, WI) [13]. The VDS cells were plated in the 6-well culture plates to 70–80% confluence (24–36 hours) prior to sample inoculation for virus isolation. The viral transport media for positive samples were thawed, clarified by brief centrifugation, and filtered through a 0.45 μm filter. Media was removed from the wells and 500 μL of inoculum were layered over the VDS cells for one hour at 37°C. Inoculums were removed and replaced with culture media and incubated at 37°C. Cells were monitored for cytopathic effect (CPE) for 1–4 days post-inoculation. At the end of day 4, culture plates were frozen and thawed once, and 500 μL of the media was used for the second passage onto another set of fresh VDS cells in a 6-well culture plates. After second passage, the culture plates were frozen and thawed once, and 50 μL of the media was subjected to nucleic acid extraction and CDV RT-PCR assay to confirm the virus isolation results.

### Sensitivity of virus isolation

Passage 3 of CDV, purchased from American Type Culture Collection (ATCC, cat #VR-1587), was used to determine the sensitivity of VDS cells for virus isolation of CDV. Ten-fold serial diluted virus were inoculated in duplicates onto the VDS cells seeded in 12 well plates (100 μL/well), cell preparation and viral isolation protocol was carried out as described above, successful virus isolation was determined by observation of CPE in the VDS cells at 1–4 days post inoculation. A 50 μL aliquot of the ten-fold serial diluted virus was subjected to nucleic acid extraction and CDV RT-PCR assay as described. A similar approach was used with the virus isolated from 3 of the study animals. Post isolation, virus was refreshed twice in new VDS cells in a T25 flask, then serially diluted and subsequently inoculated onto the VDS cells.

### Data analysis

**Fig 1:**
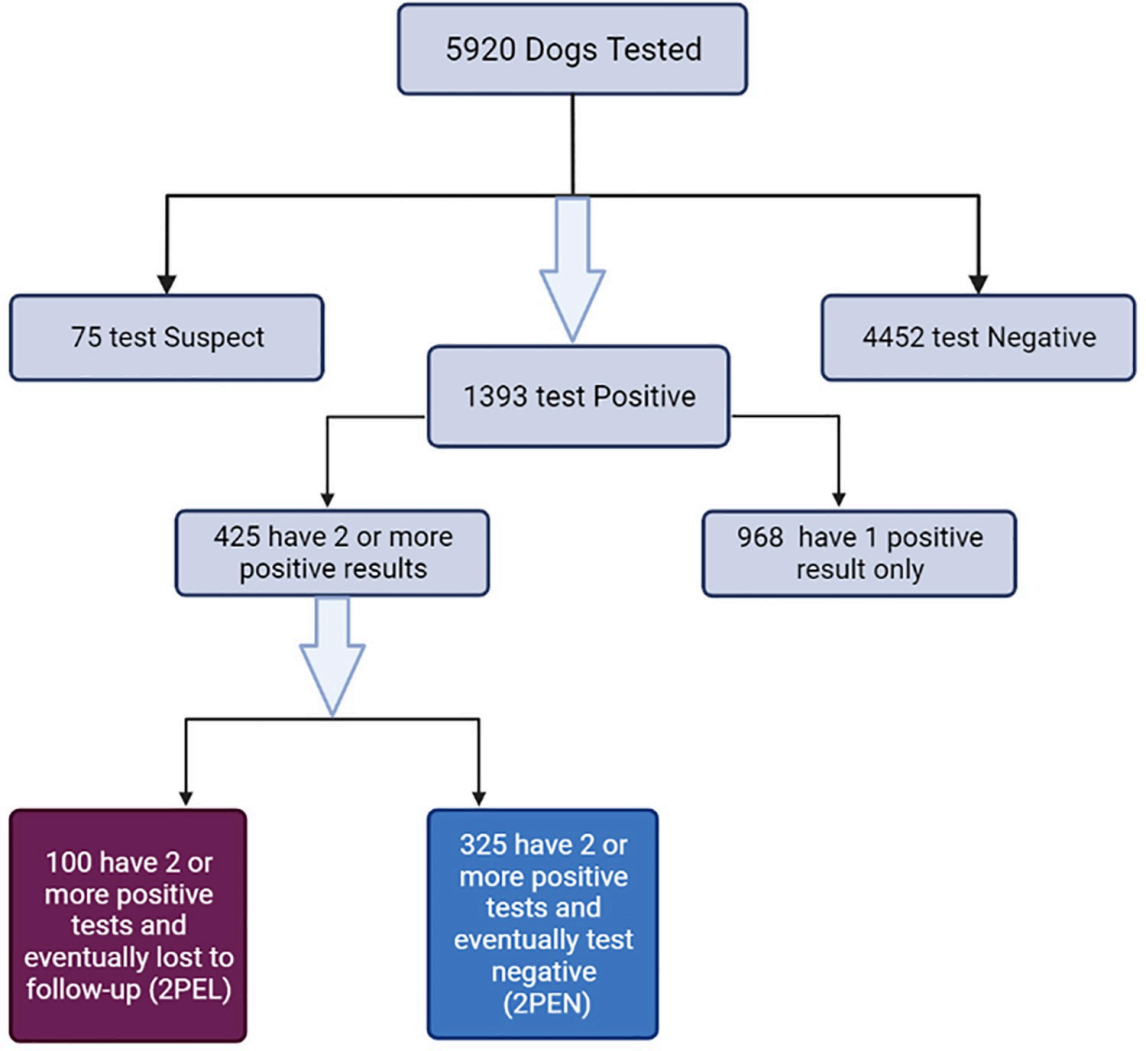
Organizational chart for RT-PCR testing in shelter dogs. 2PEL = 2 positive eventually lost; 2PEN = 2 positive eventually negative. Created with BioRender.com.

For ease of working with the data, each individual case included in the study was given a designation that described the type of data that case contained. Dogs with two or more positive results that eventually stopped shedding viral RNA were designated 2 positive eventually negative (2PEN). Dogs with two or more positive results that were eventually lost to follow up prior to a negative RT-PCR test were designated 2 positive eventually lost (2PEL). Dogs whose only datapoint was a suspect result were excluded as being neither positive nor negative (Fig 1).

## Results

### Serial RT-PCR testing

Semi-quantitative results were reported as Ct which ranged from 18.1 to 39.8 (cutoff of 40.0). A total of 5,920 dogs were tested for CDV using RT-PCR and results were as follows: 1,393 individual dogs tested positive at least once; 4,452 dogs tested negative; and 75 dogs had suspect results. The 1,393 dogs that had at least one positive test were further divided into two groups: dogs with a single positive test (n = 968) and dogs with two or more positive tests (n = 425) (range = 2–27 tests) (Fig 1).

Of these 425 dogs, 325 were followed until testing negative with no subsequent positive results and 100 dogs were eventually lost to follow up (2PEL) before testing negative with no subsequent positive results.

### Duration of positive results

**Fig 2:**
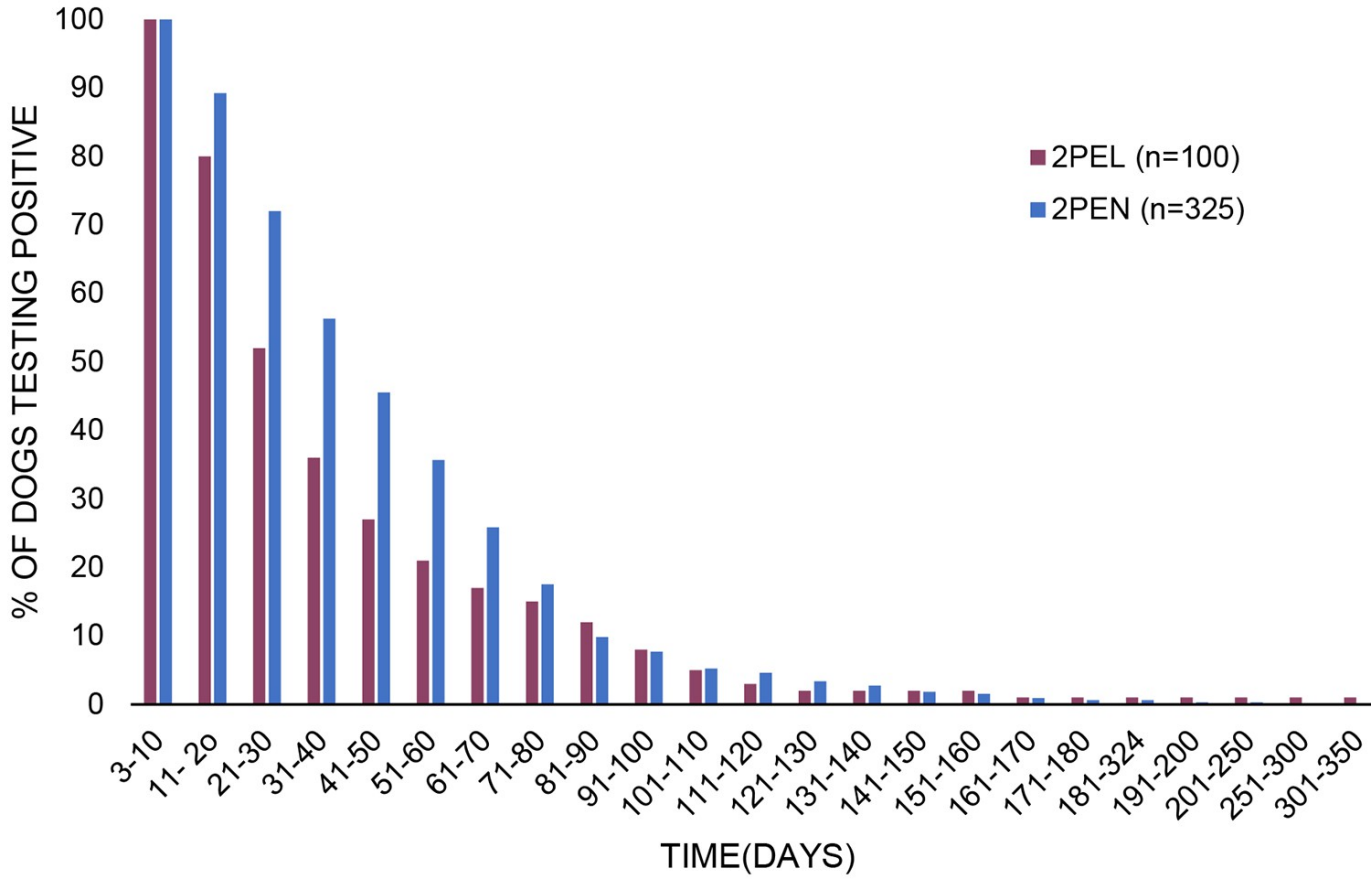
Duration of excretion of CDV RNA over time in 2PEL and 2PEN dogs. 2PEL (n = 100) indicates those dogs that had at least two positive results and were eventually lost to follow-up. 2PEN (n = 325) indicates those dogs that had at least two positive results and eventually tested negative. Samples were collected by swabbing the inside of one or both nares and/or the deep pharyngeal region.

The duration of recorded positive testing for the 2PEN and 2PEL groups combined ranged from 3 to 324 days. Because the 100 dogs in the 2PEL group were eventually lost before a negative was reported, durations described for that group underrepresent the actual full duration of positive testing. Because of this, these dogs were excluded in reporting of medians, means, and quartiles. The mean duration of positive test results for the 2PEN group, where none were lost to follow up, was 43.7 days, with a median of 34 days. 75% (245/325) of the population continued to excrete viral RNA beyond day 19 and by day 62, 25% (82/325) of the population was still shedding viral RNA (Fig 2).

### Infectious potential over time: Virus isolation

The VDS cells were highly sensitive for isolation of CDV. The CPE was readily visible as early as 24 hours post inoculation, and for most wells complete CPE was visible within 48–72 hours. There was rarely new CPE past 48 hours post-inoculation, thus making this a very efficient method for viral isolation.

Absolute quantitative method was not used to determine the sensitivity of the RT-PCR assay or the viral isolation. Rather, the sensitivity of the viral isolation method was determined qualitatively by direct comparison to RT-PCR assay. The detection limit of the published RT-PCR assay was determined as 1x 102 RNA copies/μL of template [9]. In this study, 50 μL of cultured virus was used for extraction of nucleic acid for PCR, while 100 μL of the same virus was used as inoculum in viral isolation. The detection limit of the VDS cells for CDV isolation was compared to Ct value of our RT-PCR assay for detection of the viral RNA in the inoculum. The results are reported in Table 1 and showed that virus was consistently isolated with inoculum that had a Ct of up to 37, but isolation was unsuccessful with a Ct between 38 and 40. One sample was VI positive at a dilution past a Ct of 34 while negative on RT-PCR. The direct comparison across assays confirmed the VDS cells are highly sensitive for isolation of CDV. For field samples, five times more sample (500 μL) was used for virus isolation to ensure maximum sensitivity (Table 1). The field samples used in Table 1 were from the population of dogs evaluated in this study, and they were preserved and stored using the same procedure as the samples from dogs 1–6 in the VI study.

**Table 1:** Comparison of RT-PCR and virus isolation of commercially acquired and field isolates of CDV.

	ATCC strain		Isolate 1		Isolate 2		Isolate 3	
Dilution	RT-PCR (Ct)	VI	RT-PCR (Ct)	VI	RT-PCR (Ct)	VI	RT-PCR (Ct)	VI
10^0	24.3	**POS**	22.4	**POS**	20.6	**POS**	21.2	**POS**
10^-1	27.7	**POS**	26.0	**POS**	23.2	**POS**	24.1	**POS**
10^-2	31.6	**POS**	29.5	**POS**	26.8	**POS**	27.4	**POS**
10^-3	34.9	**POS**	32.9	**POS**	30.4	**POS**	31.1	**POS**
10^-4	Neg	**POS**	37.0	**POS**	34.0	**POS**	34.5	**POS**
10^-5	Neg	Neg	Neg	Neg	38.1	Neg	39.0	Neg
10^-6	Neg	Neg	Neg	Neg	Neg	Neg	Neg	Neg
10^-7	Neg	Neg	Neg	Neg	38.2	Neg	Neg	Neg
10^-8	Neg	Neg	Neg	Neg	Neg	Neg	Neg	Neg
10^-9	Neg	Neg	Neg	Neg	Neg	Neg	Neg	Neg
10^-10	Neg	Neg	Neg	Neg	Neg	Neg	Neg	Neg

RT-PCR results are reported as cycle threshold (Ct). Virus isolation (VI) results are reported as “Pos” or “Neg” based on the presence or absence of cytopathic effect and they are located to the right of the Ct results of the same sample. One strain of CDV was from the American Type Culture Collection (ATCC) and Isolate 1-Isolate 3 were collected from shelter dogs.

### Virus isolation from field samples

**Fig 3:**
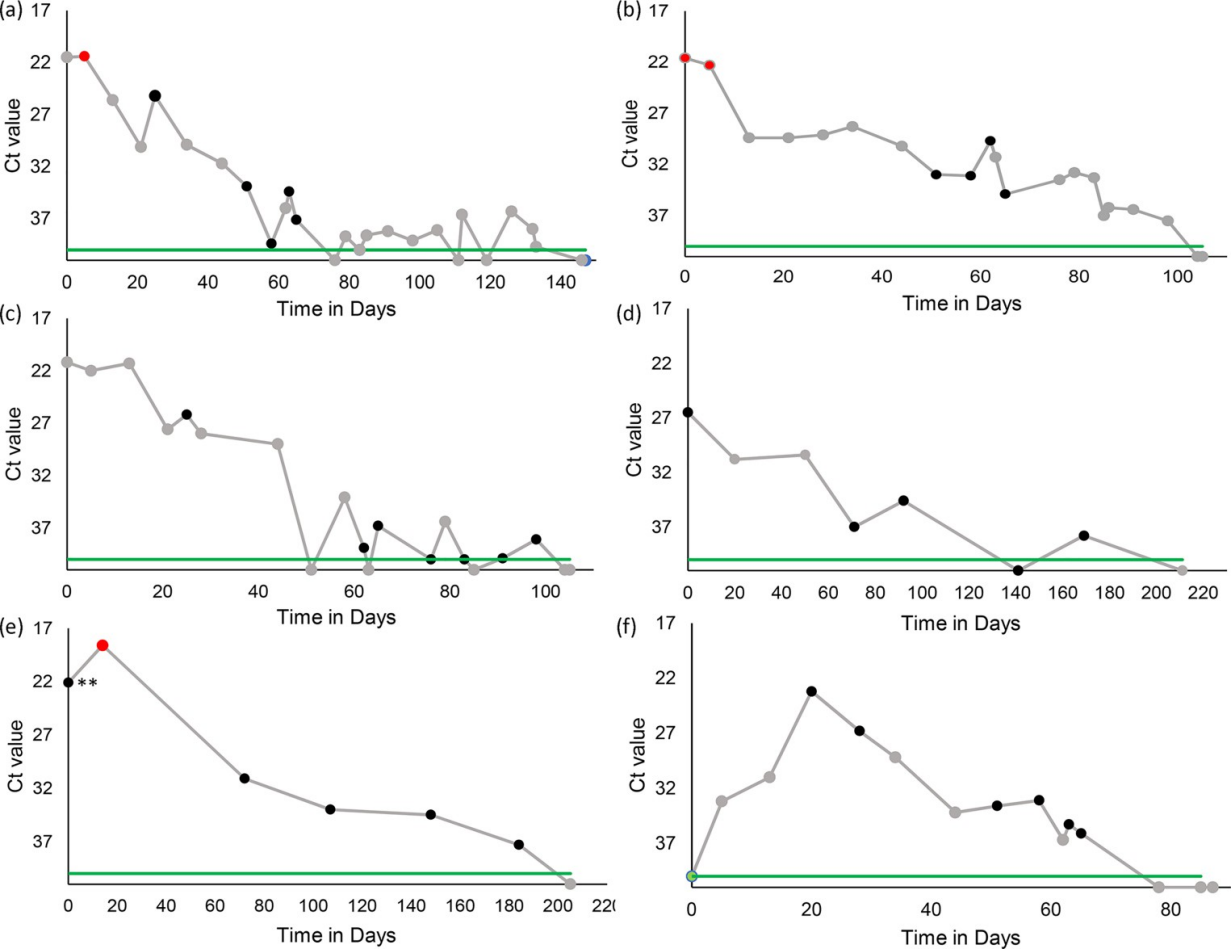
RT-PCR cycle threshold (Ct) over time(days) in six dogs. Graph (a) represents dog 1, (b) represents dog 2, (c) represents dog 3, (d) represents dog 4, (e) represents dog 5, and (f) represents dog 6. Red filled circles indicate samples where virus isolation was also positive. Black filled circles indicate samples where virus isolation was negative. Grey circles indicate samples where only RT-PCR testing was done. The green line demarcates the 40 cycle limit of detection. ** Indicates sample where the vial was broken and may have affected virus isolation results.

A series of samples from each of six of dogs (from three different animal welfare organizations) were selected for VI testing based on the established inclusion criteria. These samples were a subset of the samples described for RT-PCR testing. All six dogs were part of the 2PEN group. Each of the six dogs had between five and seven samples still available for virus isolation. Three of the six dogs had positive virus isolation results, defined by a visible CPE, during their testing periods. Viral isolation results from the nasal swab samples taken for each dog are compared side by side in Table 2. For these six dogs, the samples used for VI ranged from Day 0 to Day 184. The complete trajectory of RT-PRC results and associated VI results from the nasal swabs of each of the six dogs can be visualized in Fig 3.

**Table 2:** Virus isolation results and Ct of samples taken over time for dogs 1–6.

Day PFT	Dog 1	Dog 2	Dog 3	Dog 4	Dog 5	Dog 6
**0**	-	**21.6**	-	26.5	22.1**	-
**5**	**21.4**	**22.3**	**-**	-	-	-
**14**	-	-	-	-	**18.6**	-
**20**	-	-	-	-	-	23.2
**25**	25.2	-	26.2	-	-	-
**28**	-	-	-	-	-	26.8
**51**	33.9	33	-	-	-	33.6
**58**	39.4	33.1	-	-	-	33.1
**62**	-	29.7	38.9	-	-	-
**63**	34.4	-	-	-	-	35.3
**65**	37.1	34.9	36.8	-	-	36.1
**71**	-	-	-	37	-	-
**72**	-	-	-	-	31.1	-
**76**	-	-	Suspect	-	-	-
**83**	-	-	Suspect	-	-	-
**91**	-	-	39.9	-	-	-
**92**	-	-	-	34.6	-	-
**98**	-	-	38.1	-	-	-
**107**	-	-	-	-	34	-
**141**	-	-	-	Neg	-	-
**148**	-	-	-	-	34.5	-
**169**	-	-	-	37.8	-	-
**184**	-	-	-	-	37.3	-

Table 2 shows a comparison of virus isolation results across the samples from six dogs included in the VI study. Day PFT indicates Day Post First Test; first test is day zero. Ct values or other notes in the table represent RT-PCR results from samples from each individual dog that were available retrospectively for VI testing. Bold print (grey fill) indicates samples with positive virus isolation results. The complete trajectory of results, including VI and all RT-PCR results, for each animal, including results not shown in this table can be found in Fig 3.

** Indicates sample where the vial was broken and may have affected virus isolation results.

Positive virus isolation results were produced from samples at or near what appeared to be the peak viral RNA load recorded (lowest Ct) for each dog with Ct values between 18.6 and 22.3. The latest positive VI sample was obtained at day 14 after initiation of testing. All samples that were collected longer than 14 days after initiation of testing were negative for virus isolation.

As shown in Fig 3, dog 1 tested positive on VI only once, at the peak recorded in the series. Dog 2 tested positive at two different times during its testing window (day 0 and 5), at the peak recorded for the series and just after. Dog 5 had a negative VI on day zero, prior to peak viral load, while a subsequent sample at day 14 was positive at the peak viral load recorded in the series (lowest Ct). However, the sample on day zero for dog 5 had a tube that was broken in transport which may have affected viability (Fig 3).

## Discussion

The findings of this study suggest dogs infected with CDV may shed viable infectious virus for a much shorter time than they excrete viral RNA as detected by RT-PCR. Highly sensitive RT-PCR may continue to detect excretion of viral CDV RNA over prolonged periods of time, making it crucial to differentiate when that excretion indicates ongoing infectious risk and when the risk has ended. Waiting for a negative RT-PCR alone, or a series of two negatives, to gauge infectious risk from dogs leads to very long holding and separation periods for dogs who continue to test positive. Our findings as well as those of others also suggest that this prolonged holding is unnecessary because for each individual, the peak of viral load shedding (which will be variable for each dog) and a notable decline in shedding load in the days surrounding the peak might be used as a functional marker to gauge the end of infectious risk.

The bulk of historical studies focused primarily on making an initial diagnosis of CD rather than making attempts to identify an endpoint of infectious risk [14–16]. As treatment has become more common, finding that endpoint for dogs who have recovered clinically has become a necessity, especially for animal shelters and other population settings where welfare and lifesaving may be compromised by ongoing isolation or separation.

Veterinary textbooks have referred to CDV as being “contagious” or “excreted” for two to three months post infection but indicate that a shorter shedding period is more common [17, 18]. Studies that included virus isolation or immunofluorescent detection of viral antigen to confirm that infectious virus was present within peripheral blood cells and plasma (viremia), typically indicated a shorter period of infectiousness that ended around the time neutralizing antibodies developed (2–3 weeks post infection) [3, 15, 16, 19].

This study identifies prolonged excretion of CDV RNA as detected by RT-PCR from samples collected from a large number of dogs over the course of many years of our work managing CD in animal shelters. For dogs that had two or more positive tests, the length of time dogs remained positive for excretion of viral RNA ranged from 3–324 days. The mean duration of positive test results for the 2PEN group, where none were lost to follow up, was 43.7 days, with a median of 34 days. 75% (245/325) of the population continued to excrete viral RNA beyond day 19 and by day 62, 25% (82/325) of the population was still shedding viral RNA (Fig 2). In addition, intermittent negative results, even two negatives followed by a positive in some cases, were well demonstrated during these prolonged periods.

Similarly, Willi et al. reported on a group of 13 dogs with community derived CDV where three dogs remained positive based on RT-PCR through the fourth month of monitoring [20]. An extreme example of a long shedding dog with CDV was reported by Lanszki et al. in their 2021 manuscript describing a dog naturally infected with CDV whose urine samples tested positive by RT-PCR for 17 months following an initial positive result [21]. Holding dogs in shelters separated or in isolation for these long durations of detection of viral RNA on RT-PCR has a negative impact on disease management, animal health, welfare, and lifesaving without concrete benefit.

Closely related MV demonstrates similarly prolonged periods of viral RNA excretion detected by RT-PCR after natural infection in people and in experimental infection in primates [22, 23]. But for both CDV and MV, RNA excretion only correlates well with detection of viable infectious virus early in the course of infection.

Patients infected with MV are considered non-infectious four days after the appearance of the stereotypical maculopapular rash that occurs 10–14 days after infection because infectious virus is typically no longer detectable in blood or other samples after that time [22, 24, 25]. Viral RNA excretion for MV may remain detectable based on RT-PCR testing for long periods of time, even after viable virus is no longer detected [23, 26]. A similar phenomenon is reported with SARS-CoV-2 in people where COVID-19 patients stop shedding infectious virus early in the course of the disease but remain positive on RT-PCR testing for a much longer period [27, 28].

While dogs infected with CDV do not predictably develop a rash as people with measles do, comparing RT-PCR results with viral isolation provides insight to develop a similar marker to identify the end of the infectious period for dogs. Virus isolation has been a challenging diagnostic to utilize for CDV because while the virus is known to infect epithelial cells in vivo, when the virus is cultured in epithelial cell lines it attenuates and becomes difficult to detect because cytolytic properties are lost [29]. Over decades researchers determined primary dog macrophage and dog lymphoid cell lines were capable of cultivating the virulent form of CDV [30–32]. Seki et al. took the process an important step further in 2003 when they demonstrated that virulent CDV could be cultured in Vero cells that had been engineered to express the dog SLAM tag (VDS) [13]. Our study utilized this technique. An internal evaluation in which one purchased CDV type strain and three field sample isolates were serially diluted and reliably tested positive on VI up to a 10^-4^dilution factor, demonstrated excellent sensitivity for low viral load at RT-PCR Ct levels up to 37 (Table 1). Sensitivity was about equal to the limit of detection of RT-PCR.

Sehata et al. demonstrated, using the VDS in their experimental comparison of CDV RT-PCR RNA shedding and virus isolation, that the concentration of viable virus isolated from rectal or nasal swabs from dogs experimentally infected with CDV was at its highest on the day that the number of RNA copies detected by RT-PCR peaked, and that virus isolation results correlated well with RT-PCR results for only the two days leading up to the peak and the two days following it [33]. In our study of natural infections, viral isolation was performed on samples from six dogs who tested positive on RT-PCR for greater than 60 days. Three of the six dogs yielded positive VI results for a total of four positive samples (Table 2). The curves of RT-PCR results for the dogs whose samples had no positives on VI suggests the timing of sampling may have started after peak viral shedding. Negative VI results from samples collected post peak viral shedding are consistent and expected with what has been previously reported [33].

As in Sehata et al., virus was isolated only from samples taken near peak RNA detection and relatively early in the course of testing 14 days or less from the first positive test [33] (Fig 3). Viable virus was detected for substantially less time than the duration of positive RT-PCR results.

Just as with human measles patients, dogs infected with CDV may shed viable infectious virus for a much shorter time than they excrete viral RNA as detected by RT-PCR. Our findings and those in Sehata et al. also suggest that the peak viral load and the days surrounding it might be used as a functional marker to gauge the end of infectious risk. In shelter situations, where testing intervals are often irregular, being able to demonstrate that the viral load has declined (from peak) appears to be a marker that infectious potential has most likely passed and the dog can be considered non-infectious.

### Limitations of the study

There were several limitations in this study.

#### Dogs were tested at irregular intervals

Timing for testing was based on factors such as available staffing to collect samples, minimizing negative impact on dogs, minimizing length of stay, and maximizing efficiency of resource utilization for testing. This may have caused the durations reported to be shorter than if dogs had been tested at greater frequency.

#### Dates of exposure to CDV were unknown

Days reported are when testing occurred rather than days post exposure. Testing may have been initiated for a host of different reasons, based on clinical signs in a particular individual or as part of a response to illness in a population. Since exposure is always prior to the first positive test, data for duration may be shortened compared to experimental data in which date from known exposure can be reported.

#### Differing criteria were used for discontinuing testing

Some dogs were sampled until one negative was produced while others were sampled until two consecutive negatives were produced. Continued testing until two negatives may have revealed more prolonged excretion of viral RNA. In addition, dogs who were lost to follow-up or euthanized may have continued to test positive adding to overall means.

Although some RT-PCR positives were likely from recent vaccination on admission to a shelter, we did not attempt to differentiate underlying causes for test results that were positive for viral RNA. Because animals are thought to shed vaccine virus for only short periods of time, including dogs who may have tested positive from vaccination alone may have skewed our duration of excretion of viral RNA to be shorter than it would be if only natural infections were included [34].

As described in the selection criteria, this was a retrospective study, based on clinical results from dogs involved in outbreak settings. Only a small number of samples were still available for virus isolation testing. The testing for those six dogs did suggest similar findings as Sehata et al. and did not suggest long term infectious potential from infected dogs who continued to test positive on RT-PCR.

Testing for CDV in animal shelters is often initiated in response to clinical signs that cause suspicion for the disease or when a known or suspected exposure has occurred. The initial point of infection is often unknown. As clinical signs of CD can be subtle at first and are similar to other illnesses that are common in shelter situations, prompt identification of disease may be difficult. As a result, the timing of testing is irregular depending on veterinary input, the course that the outbreak takes, and the resources available for testing.

The irregular testing may artificially shorten the length of time that excretion of viral RNA was reported in some cases. If testing had started earlier in the course of disease, then the actual duration would be longer than reported. For dogs who were lost to follow up while testing positive, the duration of positive results also may have been longer than reported. Only the inclusion of dogs that were shedding viral RNA because of recent vaccination would likely skew the reported viral RNA excretion durations to be shortened. The prolonged periods between positives that are reported show that dogs shed for at least as long as the time period between positive test results but likely longer. It is clear that there is a subset of dogs that will shed viral RNA that is detectable by RT-PCR for prolonged periods even after VI suggests they no longer pose an infectious risk.

## Conclusions

The long duration of detection for viral RNA excretion far exceeds the duration of infectious risk. Using RT-PCR negativity as the sole definitive method for determining an endpoint for infectious risk and clearing dogs from CDV creates substantial risks to welfare, health, and life-saving for both individuals and populations. For individual animals, defining cure based on a timeframe surrounding peak viral load shedding would lead to significantly shorter isolation times allowing puppies to be socialized and adult dogs to experience a better quality of life. For animal shelters, defining a clearer and earlier point at which dogs are no longer infectious would be a critical tool for outbreak management that could save many thousands of dollars as well as many lives.

Further investigation is needed, and already underway, to clarify if the marker of viral load declining from peak as demonstrated by RT-PCR in natural infections, will correlate consistently with cessation of infectious risk. Further investigation is also needed to determine if use of such a marker is practical and leads to life saving in outbreak situations.

## Supporting information

S1 Dataset The complete minimal dataset for all dogs included in this study. (XLSX)

## References

[1] Uhl EW, Kelderhouse C, Buikstra J, Blick JP, Bolon B, Hogan RJ. New world origin of canine distemper: Interdisciplinary insights. Int J Paleopathol 2019;24:266–78. doi: 10.1016/j.ijpp.2018.12.007 30743216

[2] Appel MJ, Summers BA. Pathogenicity of morbilliviruses for terrestrial carnivores. Veterinary Microbiology 1995;44:187–91. doi: 10.1016/0378-1135(95)00011-x 8588312

[3] Appel MJG. Pathogenesis of canine distemper. Amer J Vet Res 1969;30:1167–82. 4894003

[4] Cornwell HJC, Campbell RSF, Vastis JT, Penny W. Studies in Experimental Canine Distemper II. Virology, Inclusion Body Studies, and Haematology. J Comp Path 1965;75:19–34.

[5] Tatsuo H, Ono N, Yanagi Y. Morbilliviruses Use Signaling Lymphocyte Activation Molecules (CD150) as Cellular Receptors. J Virol 2001;75:5842–50. doi: 10.1128/JVI.75.13.5842-5850.2001 11390585 PMC114299

[6] Sawatsky B, Wong X-X, Hinkelmann S, Cattaneo R, von Messling V. Canine Distemper Virus Epithelial Cell Infection Is Required for Clinical Disease but Not for Immunosuppression. J Virol 2012;86:3658–66. doi: 10.1128/JVI.06414-11 22278252 PMC3302517

[7] Pratakpiriya W, Ping Teh AP, Radtanakatikanon A, Pirarat N, Thi Lan N, Takeda M, et al. Expression of canine distemper virus receptor nectin-4 in the central nervous system of dogs. Sci Rep 2017;7:349. doi: 10.1038/s41598-017-00375-6 28336928 PMC5428276

[8] Miller L, Zawistowski S, Zawistowski S. Shelter Medicine for Veterinarians and Staff. Hoboken, UNITED STATES: John Wiley & Sons, Incorporated; 2013.

[9] Tests and Services | IDEXX Reference Laboratories—IDEXX US n.d. https://www.idexx.com/en/veterinary/reference-laboratories/tests-and-services / (accessed March 12, 2022).

[10] Elia G, Decaro N, Martella V, Cirone F, Lucente MS, Lorusso E, et al. Detection of canine distemper virus in dogs by real-time RT-PCR. J Virol Methods 2006;136:171–6. doi: 10.1016/j.jviromet.2006.05.004 16750863

[11] Lin W-HW, Kouyos RD, Adams RJ, Grenfell BT, Griffin DE. Prolonged persistence of measles virus RNA is characteristic of primary infection dynamics. Proc Natl Acad Sci U S A 2012;109:14989–94. doi: 10.1073/pnas.1211138109 22872860 PMC3443140

[12] Chirathaworn C, Sripramote M, Chalongviriyalert P, Jirajariyavej S, Kiatpanabhikul P, Saiyarin J, et al. SARS-CoV-2 RNA shedding in recovered COVID-19 cases and the presence of antibodies against SARS-CoV-2 in recovered COVID-19 cases and close contacts, Thailand, April-June 2020. PLOS ONE 2020;15:e0236905. doi: 10.1371/journal.pone.0236905 33119712 PMC7595404

[13] Seki F, Ono N, Yamaguchi R, Yanagi Y. Efficient Isolation of Wild Strains of Canine Distemper Virus in Vero Cells Expressing Canine SLAM (CD150) and Their Adaptability to Marmoset B95a Cells. J Virol 2003;77:9943–50. doi: 10.1128/jvi.77.18.9943-9950.2003 12941904 PMC224612

[14] Haines DM, Martin KM, Chelack BJ, Sargent RA, Outerbridge CA, Clark EG. Immunohistochemical Detection of Canine Distemper Virus in Haired Skin, Nasal Mucosa, and Footpad Epithelium: A Method for Antemortem Diagnosis of Infection. J VET Diagn Invest 1999;11:396–9. doi: 10.1177/104063879901100502 12968751

[15] Brown RALl, Morrow A, Heron I, Chong SN. Immunocytological confirmation of a diagnosis of canine distemper using cells in urine. J Small Anim Pract 1987;28:845–51. 10.1111/j.1748-5827.1987.tb01351.x.

[16] Fairchild GA, Wyman M, Donovan EF. Fluorescent antibody technique as a diagnostic test for canine distemper infection: detection of viral antigen in epithelial tissues of experimentally infected dogs. Am J Vet Res 1967;28:761–8. 5340996

[17] Vahlenkamp TW. Canine distemper and other viral infections. In: Ettinger SJ, Feldman EC, Côté E, editors. Textbook of Veterinary Internal Medicine. 8th ed., St. Louis (MO): Elsevier; 2017, p. 1006–12.

[18] Greene C, Vandevelde M. Canine distemper. In: Greene C, Sykes J, editors. Infectious diseases of the dog and cat. 4th ed., Philidelphia (PA): Saunders; 2011, p. 25–42.

[19] Krakowka S, Higgins RJ, Metzler AE. Plasma phase viremia in canine distemper virus infection. Am J Vet Res 1980;41:144–6. 7362119

[20] Willi B, Spiri AM, Meli ML, Grimm F, Beatrice L, Riond B, et al. Clinical and molecular investigation of a canine distemper outbreak and vector-borne infections in a group of rescue dogs imported from Hungary to Switzerland. BMC Vet Res 2015;11:154. doi: 10.1186/s12917-015-0471-0 26179635 PMC4504088

[21] Lanszki Z, Zana B, Zeghbib S, Jakab F, Szabó N, Kemenesi G. Prolonged Infection of canine distemper virus in a mixed-breed dog. Vet Sci 2021;8. 10.3390/vetsci8040061. PMC8069365 33920469

[22] Permar SR, Klumpp SA, Mansfield KG, Kim W-K, Gorgone DA, Lifton MA, et al. Role of CD8+ Lymphocytes in Control and Clearance of Measles Virus Infection of Rhesus Monkeys. J Virol 2003;77:4396–400. doi: 10.1128/jvi.77.7.4396-4400.2003 12634396 PMC150640

[23] Griffin DE, Lin W-H, Pan C-H. Measles virus, immune control and persistence. FEMS Microbiol Rev 2012;36:649–62. doi: 10.1111/j.1574-6976.2012.00330.x 22316382 PMC3319515

[24] Healthcare Professionals: Clinical Resources. Centers for Disease Control and Prevention 2021. https://www.cdc.gov/measles/hcp/index.html (accessed October 21, 2021).

[25] Ruckle G, Rogers KD. Studies with Measles Virus: II. Isolation of Virus and Immunologic Studies in Persons Who Have Had the Natural Disease. J Immunol 1957;78:341–55. 13449323

[26] Riddell MA, Moss WJ, Hauer D, Monze M, Griffin DE. Slow clearance of measles virus RNA after acute infection. Journal of Clinical Virology 2007;39:312–7. doi: 10.1016/j.jcv.2007.05.006 17625962

[27] Nomura T, Kitagawa H, Omori K, Shigemoto N, Kakimoto M, Nazmul T, et al. Duration of infectious virus shedding in patients with severe coronavirus disease 2019 who required mechanical ventilation. J Infect Chemother 2022;28:19–23. doi: 10.1016/j.jiac.2021.09.006 34538728 PMC8429366

[28] Widders A, Broom A, Broom J. SARS-CoV-2: The viral shedding vs infectivity dilemma. Infect Dis Health 2020;25:210–5. doi: 10.1016/j.idh.2020.05.002 32473952 PMC7237903

[29] Appel MJ. Reversion to virulence of attenuated canine distemper virus in vivo and in vitro. J Gen Virol 1978;41:385–93.

[30] Appel MJG, Pearce-Kelling S, Summers BA. Dog Lymphocyte Cultures Facilitate the Isolation and Growth of Virulent Canine Distemper Virus. J VET Diagn Invest 1992;4:258–63. doi: 10.1177/104063879200400306 1387554

[31] Friedlander JM, Summers BA, Appel MJG. Persistence of virulent canine distemper virus in lymphoblastoid cell lines. Archives of Virology 1985;86:47–62. doi: 10.1007/BF01314113 2994601

[32] Appel MJ, Jones OR. Use of alveolar macrophages for cultivation of canine distemper virus. Proc Soc Exp Biol Med 1967;126:571–4. doi: 10.3181/00379727-126-32509 6079942

[33] Sehata G, Sato H, Ito T, Imaizumi Y, Noro T, Oishi E. Use of quantitative real-time RT-PCR to investigate the correlation between viremia and viral shedding of canine distemper virus, and infection outcomes in experimentally infected dogs. J Vet Med Sci 2015;77:851–5. doi: 10.1292/jvms.14-0066 25728411 PMC4527509

[34] Burton JH, Veir JK, Pearce L, Hawley JR, Lappin MR. Detection of canine distemper virus RNA vaccine. In: Research Abstract Program of the 26th Annual ACVIM Forum San Antonio, TX, June 4-June 7, 2008. J Vet Intern Med. 2008 May-Jun; 22(3): 703. doi: 10.1111/j.1939-1676.2008.0103.x Epub 2008 Jul 10. PMCID: PMC7166787.

